# The Simple Treatment of Disease Deduced from the Methods of Expectancy and Revulsion

**Published:** 1842-10-01

**Authors:** 


### The Simple Treatment of Disease deduced from the Methods of Expectancy and Revulsion. By *James M. Gully*, M.D. Churchill, 1842.

This is a very curious and learned work—one half of which is occupied with the opinions and practices of all the principal Medici from Hippocrates downwards, as to the methods of expectancy and revulsion in the treatment of diseases. Over this wide field we certainly shall not wade. We think our time may be more usefully employed otherwise. In the third part or chapter, Dr. Gully makes his choice *from* rather than *between* the two rival doctrines above alluded to. Our author offers a few physiological observations on the organs and laws of animal and organic life, which we need not notice here, as they are familiar to every medical man. It appears to be Dr. Gully's conviction that most of our diseases commence in the organs of vegetative life (the digestive organs for example) and thence radiate to the organs of animal life, as the brain, spine and nerves. "But while this fact is undoubted, it is equally certain that disease much more generally begins in the vegetative, and is propagated to the animal organs, than the contrary. Commence where it may, however, the ultimate aim of medical treatment is to preserve the life of the animal." In this sentiment we fully agree—and we believe there would not be found a dissentient voice between the North and South Poles.

But disease may and does begin occasionally in the organs of animal life.

"Turning to the instances in which disease commences in the organs of animal life, we find, as might be expected from the preceding details, indications of much more pressing and immediate mischief. For in this case the citadel of man's sentient and percipient being is attacked directly, and, if it be vehemently assaulted, crushes his vegetative nature in its fall. A strong apoplectic seizure, the effusion of serous fluid on the brain as a consequence of its acute inflammation, the inflammation itself, the concussion of the brain substance by violence or its sudden compression by fracture of the skull, may any of them in a very brief space extinguish both animal and vegetative life. The chronic forms of brain disease tend to the same end, if means are not taken to arrest them : and softening of the cerebral substance, gradual accumulation of fluid, serum, or pus, on its surface or in its cavities, effect as certainly what the acute morbid states above mentioned do with greater rapidity." 99.

Dr. Gully comes to the conclusion "*that no one sinks under disease until it has invaded the viscera of vegetative and animal life.*" Dr. G. acknowledges that this is a trite truth. Macbeth maintained that this aphorism held good *before his day.*

" Time *was*
That when the brains were out, the man would die."

But, to his astonishment and dismay, he found that Old Duncan was not obedient to this law of Nature ! The following quotation will shew our author's drift in the foregoing passage.

"Palpable as this truth may appear, and trite though it be pronounced, the frequency with which it is placed out of view in the treatment of disease, is for that very reason the more astounding. It matters little to a vast number of practitioners where disorder commences, these same viscera, on whose integrity life depends, are made, in every case, the battle-field for the operations of powerful and conflicting agents. Take first the cases of disease wherein the sympathies between the external surface and the contents of the abdomen, and between the latter, are morbidly excited.

Individuals receive wounds on, or are attacked by inflammations of, the limbs, and are said to die of them; the efficient cause of death, mean-time, being the extension of irritative action from the surface to the vegetative viscera, and thence to the brain. Is this fact, constantly and clearly before the mental vision of him who by harsh medication—by large doses of mercurials, drastic purgatives, and stimulants—actually predisposes the viscera to receive the irritation from without ? Does it occur to him that the fever which supervenes on some, it may be, slight wound, is in great part attributable to the artificial irritation which his applications to the sentient and highly-sympathising stomach and bowels have produced ?

After a longer or shorter period of malaise, a general fever breaks out in an individual, and all the symptoms announce an irritated condition of the vegetative viscera, the tongue, stomach, bowels, kidneys, heart and lungs, being all disordered in function. When to these organs thus situated the same harsh medication is applied, can it be said that the fact that *death comes by the viscera* is duly appreciated ? And when, in spite of, or rather in consequence of, such treatment, the brain becomes affected with delirium and then stupor, can any one, having that fact before him, fail to perceive the consequence alluded to ?

In truth, the viscera are too often reputed only as vehicles for the reception of medicines and as localities for the erection of revulsive action. Nor is this latter use always an abuse, as I shall have occasion presently to show. But I maintain that in the instances just referred to, and indeed in all cases wherein the sympathies between the external surface and the viscera of vegetative life, and those between these last, are for the time alone involved, the plain and positive indications of nature are in direct opposition to the establishment of any irritation by medicinal means in those viscera. These are already in a state of derangement, and should be exempted from agents which further perturb their functions." 103.

Dr. Gully goes on to exemplify these principles. Thus in acute or inflammatory dyspepsia, the symptoms of which we need not describe, our author tells us that the horizontal position and plenty of cold water will cure the disease in two or three days. No aperient medicine need be taken —no leeches applied—no small doses of blue-pill—no counter-irritation, except that of a lavement, are at all necessary.

"Medicine by the mouth is worse than useless in such cases as these: it is positively hurtful. It *relieves* in half the time the above simple treatment requires. The difference is, that the latter *cures*, allows the patient to rise up well, eat his ordinary food, and pursue his ordinary avocations, without the immediate distress, without the certain relapse and consequent necessity for recurrence to the 'blue pill and black draught,' which attend the treatment by drugs." 107.

The treatment of chronic dyspepsia is only a modification of the above. A little more exercise is allowed—"small but frequent draughts of cold water are also highly beneficial, and, conjoined with revulsion on the skin of the abdomen, will generally supersede the use of cathartics."

"Rest of limb is likewise imperative in the majority of cases of this sort, and exertion is found to be very distressing. The sinking and gnawing of the stomach, so usually mistaken for appetite, and appeased by food, will invariably disappear in the recumbent posture, and after a draught of cold water. On the other hand, it comes on when the limbs are employed, especially when the stomach is empty. When, however, the diseased stomach has involved the liver in mischief, and the symptoms denote congestion of that organ, it becomes necessary to expedite the circulation there. This is vulgarly done by mercurials: but a much safer mode is to employ counter-irritating frictions over the region of the liver, and carefully (with reference to the sympathies between the stomach and the seat of voluntary power, the brain and spinal cord) apportion exercise of the muscles in riding or walking." 110.

We shall only offer one more extract to shew our author's horror of all active treatment, even in febrile and inflammatory affections—those of the head excepted.

"With this view of the febrile condition, (and it is one for the reasonableness of which eminent names in medicine might be cited,) the propriety of violent revulsion practised on the organs of vegetative life is, at least, very problematical. The last are labouring to effect relief for themselves by transferring the irritative action to one of the great emunctories, the lower bowel, the kidneys, or the skin, —to the latter in the great majority of cases. How is this to be done whilst means are taken to retain the irritative action in themselves? whilst calomel and antimony are exciting the stomach, and senna, scammony, and other strong purgatives, are drawing blood to the whole canal to supply the enormous excretions they produce? whilst, as if to *force* all the emunctories together, a conflict of diuretics and sudorifics comes to aid the mercurials and purgatives in making 'confusion worse confounded' in the already oppressed and irritated internal organs? It is such treatment as this that justifies the jest passed on medicine in the definition of a physician as one who, armed with certain weapons, lays about him in all directions, with at least an equal chance of extinguishing the patient as the disease. For, although the patient may recover, notwithstanding the tumult into which the viscera have been thrown, the chance is considerably in favour of this tumult being extended to the great viscus of animal life, the brain, whose function is first deteriorated and then destroyed." 116.

This comes of our youth imbibing early the gum-water and eau sucré treatment of the continental physicians! From shuddering at the extremes of the calomel and black-draught practices, they fly to the antipodes of physic and do nothing.

This is one of the worst evils attending an early medical education on the Continent. Neither the students nor their masters remember that the chylopoietics of a Frenchman and a German are habitually corroded by sour wine and sourer krout, and will not bear the chologogues and hydrogogues so useful in England.

At the same time, we are ready to acknowledge that the author of this little work, though strongly tinctured with the Hippocratic expectancy, and the Gallo-Germanic hydrargyro and catharso-phobia, has set forth very many judicious cautions and excellent pieces of advice, that are well deserving the attention of some of our creosoting, chalybeating, thunderbolting, phlebotomising, and Augean-stable-cleansing HEROES of the present day.

P.S.—Is Dr. Gully a HYDROPATH at heart?

